# Calibration and Verification of Dynamic Particle Flow Parameters by the Back-Propagation Neural Network Based on the Genetic Algorithm: Recycled Polyurethane Powder

**DOI:** 10.3390/ma12203350

**Published:** 2019-10-14

**Authors:** Ping He, Yiwei Fan, Banglong Pan, Yinfeng Zhu, Jing Liu, Darong Zhu

**Affiliations:** College of Mechanical and Electrical Engineering, Anhui Jianzhu University, Hefei 230601, China; panbanglong@ahjzu.edu.cn (B.P.); zhuyinfeng73@163.com (Y.Z.); liujing@ahjzu.edu.cn (J.L.); zhudarong@ahjzu.edu.cn (D.Z.)

**Keywords:** discrete element method, ultrafine agglomerated powder, dynamic angle of repose, predictive model, mean-shift clustering analysis

## Abstract

The discrete element method (DEM) is commonly used to study various powders in motion during transportation, screening, mixing, etc.; this requires several microscopic parameters to characterize the complex mechanical behavior of the particles. Herein, a new discrete element parameter calibration method is proposed to calibrate the ultrafine agglomerated powder (recycled polyurethane powder). Optimal Latin hypercube sampling and virtual simulation experiments were conducted using the commercial DEM software; the microscopic variables included the static friction coefficient between the particles, collision recovery coefficient, Johnson–Kendall–Roberts surface energy, static friction coefficient between the particles and wall, and collision recovery coefficient. A predictive model based on genetic-algorithm-optimized feedforward neural network (back propagation) was developed to calibrate the microscopic DEM simulation parameters. The cycle search algorithm and mean-shift cluster analysis were used to confirm the input parameters’ range by comparing the mean value of the dynamic angle of repose measured via the batch accumulation test. These parameters were verified by the baffle lifting method and the rotating drum method. This calibration method, once successfully developed, will be suitable for use in a variety of fine viscous powder dynamic flow conditions.

## 1. Introduction

Currently, the main methods that are used for recovering and recycling thermosetting plastic waste include the mechanical and physical method, the chemical method, and the energy recovery method. The mechanical and physical method [1,2] can achieve complete recovery with high recovery efficiency and low pollution. The recycling method is based on the principle that materials accumulate under various mechanical forces, causing the accumulation of mechanical energy, destroying the crosslinked molecular structural network, generating a recycled powder with a low crosslinking degree, and realizing the recovery and recycling of the thermosetting plastic [3]. 

**Figure 1:**
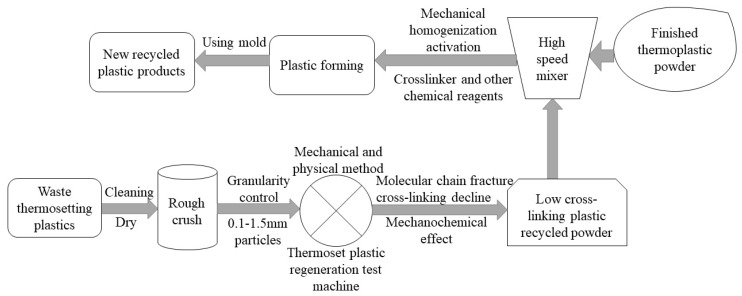
Recycling route of the thermosetting plastics based on the mechanical and physical method.

In industrial production, as shown in Figure 1, in the process of mixing and regenerating the material after mechanical force pulverization, the active regenerated material is usually added, mixed, and regenerated; then, a reagent such as a crosslinking agent is added and solidified to form a thermoplastic finished material. However, during the production process, the powder is often in motion owing to processes such as transportation, sieving, and mixing. Therefore, during the DEM calibration simulation, it is necessary to use the calibration test under complex dynamic conditions to characterize the actual mechanical behavior of the powder.

Recently, the discrete element method (DEM) has become a popular tool for describing the movement of particles, including powders, seeds, and soil, especially in the mining, chemical, pharmaceutical, and environmental industries, with the improvement of the computer capabilities [4,5]. DEM has been demonstrated to be a better tool than other simulation methods at this stage because it can analyze and optimize the process parameters associated with the preparation of the particle material and the operation of equipment used in the industry. In DEM simulations, several physical properties of the materials involved in collision and the contacts that occur between them are readily available, including the parameters that describe the intrinsic properties of a single contact material such as its density, size, Poisson’s ratio, and Young’s modulus. However, the contact characteristics will depend upon the specific model that is used while describing the interaction between particles and between a particle and the device geometry. The commonly used contact models are the Hertz–Mindlin, Johnson–Kendall–Roberts (JKR), and Bonding models; however, these models require the calibration of a large number of contacts. Further, the parameters can be used to accurately characterize the actual working conditions [6,7]. In the DEM parameter calibration test, the application of direct measurement calibration or indirect virtual calibration is the primary method for obtaining the aforementioned parameters. The characteristic parameters that are easy to measure can be directly measured experimentally, and the measurement results can be considered to be the input value for the DEM parameters. However, because of the anisotropy of some particulate matter, the direct measurement results are observed to vary considerably. The difference between the established particle model and the actual model is large, and the parameter measurement value cannot be directly applied to the DEM numerical simulation. Therefore, numerous researchers have proposed the usage of virtual simulation experiments to calibrate the contact parameters required in discrete element simulations and have conducted extensive research [8,9]; however, these calibrations mostly use the “testing method”, which exhibits high computing costs. To solve this problem, researchers have attempted to use predictive models or apply optimization algorithms. Consider Wu [10] as an example, the triaxial shear tester and stress–strain curve were used to determine the macroscopic mechanical properties of the soil. Then, the genetic neural network was used to derive the DEM parameters. Cheng [11] proposed a sequential quasi-Monte Carlo filter as a new calibration method for the DEM models of granular materials. Rackl and Hanley [12] used Latin hypercube sampling and the kriging method to describe and demonstrate a method based on the static angle method. Zhou et al. [13] calibrated the irregularly expanded graphite particles, combining optimal Latin hypercube sampling and virtual simulation experiments to obtain the DEM parameters that are to be calibrated using an adaptive simulated annealing optimization algorithm; further, the membership function was selected. The output values were used to obtain several sets of optimal contact parameter sets. Do et al. [14] developed a parameter calibration framework for discrete element models based on a multi-objective genetic algorithm (GA). They used quartz sand particles to optimize the model accuracy and simulation time and demonstrated that the contact parameters of the materials can be accurately calibrated within the optimal simulation time. Simultaneously, coarse granulation is the most common modeling approach for the calibration of the discrete element parameters of special particles (including wet particles and ultrafine polymer powders), as demonstrated by Alizadeh et al. [15] for polyethylene glycol 400 (PEG 400). In a study investigating the coating treatment of the EP particles, the concept of cohesive number was used in the DEM numerical simulation to measure the shear modulus of the material or to change the particle size to ensure that the surface energy could be rapidly scaled and that the calibration time could be substantially reduced. Nasato et al. [16] used DEM to numerically simulate the wet granulation of continuous granulators and investigated the possibility of seed-particle formation during a continuous process to reduce the number of trials and processes. Further, a control method was designed to define dimensionless number (cohesion number) on the basis of the effect of particle cohesion and gravitational potential energy to investigate the effects of factors, such as the drum rotation speed, particle surface energy, and particle size ratio, on the performance of the seed granulator. Among them, reducing the shear modulus of the particles and improving the calculation ability are also necessary content for coarse grain modeling. For example, J. Hærvig et al. [17] gave an analytical derivation criterion for reducing computation time by reducing particle stiffness. The validity of the criterion was verified by comparing the experimental data with the particle simulation of reducing the particle stiffness. Yile Gu et al. [18] used an improved cohesion model for fluidized CFD-DEM simulations. For viscous particles, the predicted flow pattern depends on the value of the particle spring stiffness used in the simulation. An improved cohesive model was proposed, which was verified by the simulation of two-particle collision, which provides satisfactory results for the fluidization of particles.

**Figure 2:**
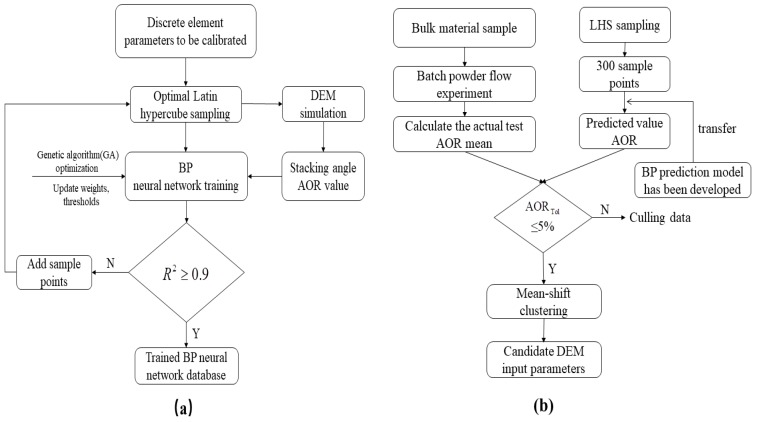
Discrete element parameter calibration process: (**a**) development and (**b**) application of the back-propagation (BP) neural network.

Therefore, based on the aforementioned summary and given that the recycled polyurethane (PU) powder investigated in this study also exhibits strong adhesion, we selected the JKR model exhibiting the agglomeration bonding effect as the characterization method to select the most sensitive discrete meta-contact parameters. Simultaneously, we used the sample space obtained after Latin hypercube sampling (LHS) sampling to simulate a series of parameter values on the basis of the determined range of the contact parameters via the commercial DEM software. Finally, the back-propagation (BP) neural network is trained to obtain an accurate prediction model. The calibration process is divided into two phases. The first phase is based on the development of a BP neural network prediction model optimized by the GA (Figure 2a), whereas the second phase is based on a neural network model developed for specific particulate materials. The application of the calibration experiment resulted in the selection of the DEM parameters (Figure 2b).

Training a BP neural network requires a series of DEM simulations in the selected range of simulation parameters. Herein, the BP neural network was trained in conjunction with the improved FBS-104 powder meter test. In engineering applications [19], the correlation coefficient (R^2^) is usually greater than 90% before the neural network can be used as the predictive model; thus, the correlation coefficient can be considered to be an evaluation criterion. Figure 2b denotes the application of the BP neural network after training. The same powder measuring device is used to test the PU sample to avoid introducing an error into the prediction model. Based on the trained BP neural network database, the output will be the angle of repose, which is input into the loop search algorithm, and the mean-shift cluster analysis method is used to solve the feasible domain of the DEM parameters. Then, the candidate DEM parameter set are determined from the database and verified using the baffle lift and rotary drum methods. This calibration method, once successfully developed, will be suitable for use in a variety of fine viscous powder dynamic flow conditions.

## 2. Experimental Method and DEM Modeling

### 2.1. Funnel Flow Meter Test Device

**Figure 3:**
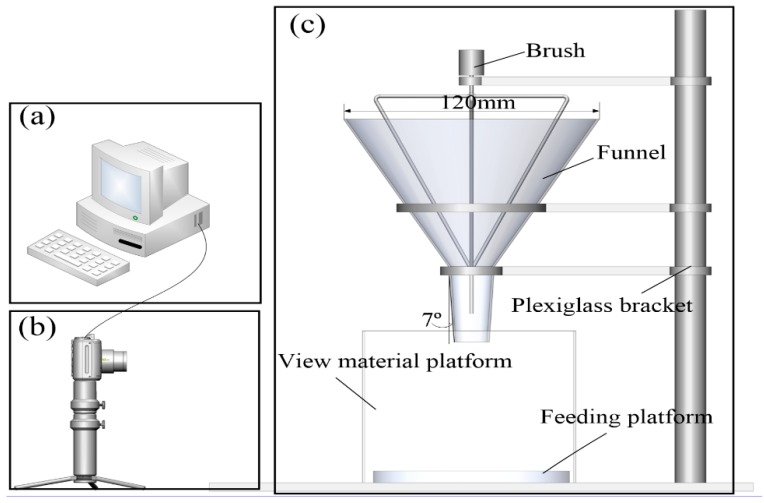
Static angle acquisition process used in the stacking tests: (**a**) image processing; (**b**) data collection; and (**c**) measurement using the improved FBS-104 powder measuring instrument.

The test material was obtained by pulverizing the thermosetting PU closed rigid foam plastic in a homemade thermosetting plastic pulverization and reclaiming test machine; powders with particle sizes of 80 (0.18 mm) and 120 mesh (0.125 mm) and a small amount of 200-mesh (0.0750 mm) powder were obtained. During the calibration process, the PU powder of the calibration object is prone to agglomeration; therefore, agglomeration occurs in the rotating drum, leading to the formation of a dynamic accumulation angle and the occurrence of the “avalanche” phenomenon, which is difficult to overcome [20]. Therefore, the use of a funnel flow meter (modified FBS-104 powder meter) can improve the cumulative mechanical properties caused by the angle. The device comprised a 120-mm funnel, 94-mm diameter circular loading platform, brush, and Plexiglas bracket (Figure 3). However, the powder cannot be normally formed to reduce the “pour” phenomenon of the powder particles during the free-fall movement of the PU powder. The funnel outlet was designed to have a small 7° angle of inclination to reduce the material drop rate. During the whole test process, the material slides down along the funnel wall with the brush at 60 rpm. Finally, the angle of repose of the powder particles can be measured when the powder to be discharged accumulates on the receiving table to form a stable material pile.

### 2.2. Angle-of-Repose Measurement

**Figure 4:**
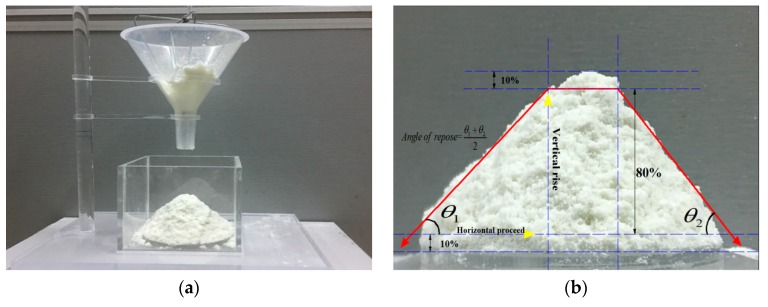

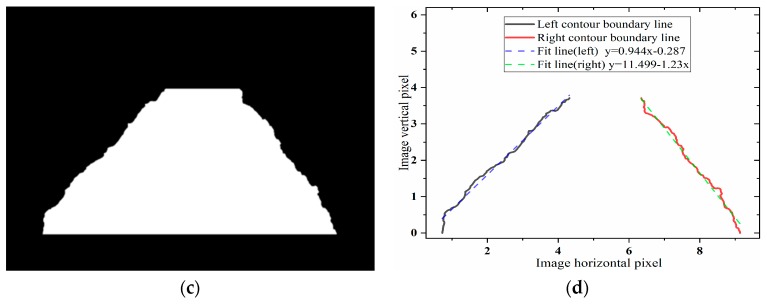
Angle-of-repose measurement process: (**a**) stacking experiment; (**b**) original image; (**c**) binarized image; and (**d**) boundary fit.

The angle of repose is used to describe the application of material particles in specific scenarios and the corresponding mechanical behavior, including the liquid bridge force, friction force, van der Waals force, and other forces that often exist between particles. Therefore, the material particles are studied by considering the angle of repose as a macroscopic response value to ensure that the appropriate microscopic DEM parameters of the material can be indirectly identified based on these response values. The angle of repose is most commonly measured with a material in its unconstrained state, as depicted in Figure 4a; the material particles are stacked and formed on the horizontal surface without collapse [21], and the slope of the formed material is observed, which can be converted into the material accumulation angle.

Figure 4b–d denote that the PU powder pile exhibits good symmetry. Alizadeh et al. [15] proposed that the high material is generally 10%–90% to avoid large fluctuations in the boundary contour of the material particles during stacking. For fitting the final boundary contour, the contour is read using the computer image processing technology and then processed by gradation and binarization; the boundary is subsequently extracted, and the boundary point is linearly fitted using the least-squares method to obtain the left and right boundaries of the powder particle stack. The equation of the contour is linearly fit, and the average value measured using the left and right angles is the angle of repose. The specific process is presented in Figure 4a–d.

### 2.3. DEM Virtual Test Model

#### 2.3.1. Coarse-Grain Modeling of the Particle Discrete Element Based on the JKR Contact Model

**Figure 5:**
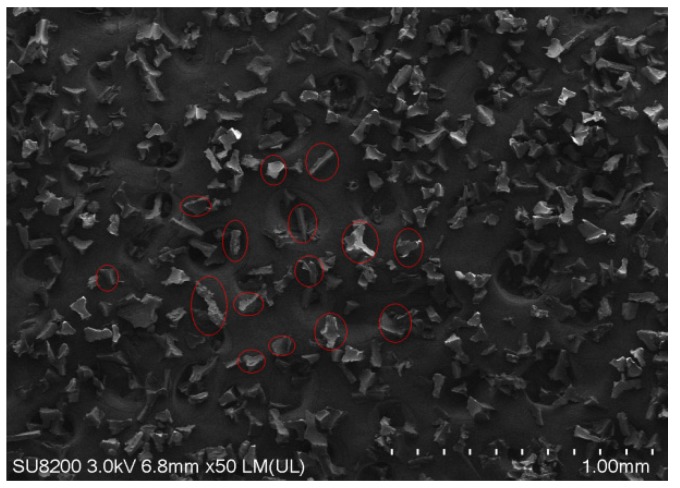
Microstructure of the 120-mesh polyurethane powder.

**Figure 6:**
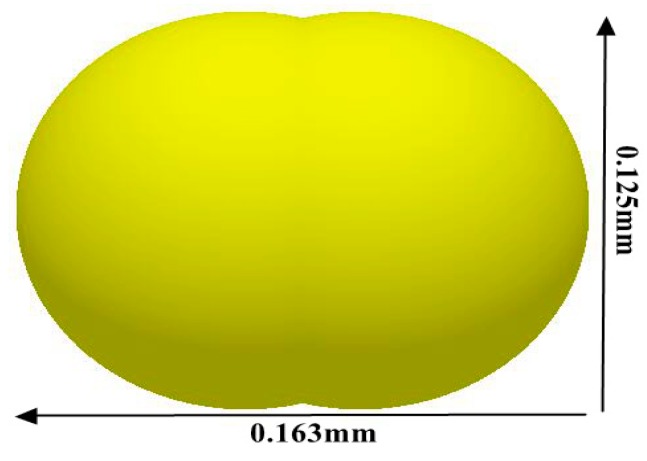
Approximate replacement model for polyurethane powder (120 mesh) during the simulation.

In this calibration test, we mainly studied a thermosetting PU powder with a particle size of 120 mesh (0.125 mm). A scanning electron microscopy image of the powder is depicted in Figure 5, denoting that the powder particles are mostly not spherical. Therefore, the mechanical interlocking of the particles may substantially contribute to the volumetric shear strength of the particulate material and may affect its fluidity. Härtl and Ooi [22] denoted that geometric interlocking of the nonspherical particles strongly affects the in vivo friction of the particulate materials and should be introduced in the DEM simulations to capture the volumetric shear strength of the material. Simultaneously, Bharadwaj et al. [23] proposed that duplicating the exact shape of the particles is unnecessary for calibrating the powder discrete element parameters; they observed that the use of double-sphere paired particles with an aspect ratio of greater than 1 can provide a satisfactory quantitative prediction and that the aspect ratio could be increased to 2 without resulting in any significant difference. Given that the PU powder used in this study exhibits a relatively complicated mechanical behavior and avoids large errors associated with the establishment of the DEM model, it is further verified in Section 2.3.3. Thus, we can conclude that the ball pairing with an aspect ratio of 1.3 was appropriate. This model is a suitable replacement for the PU powder (Figure 6).

**Figure 7:**
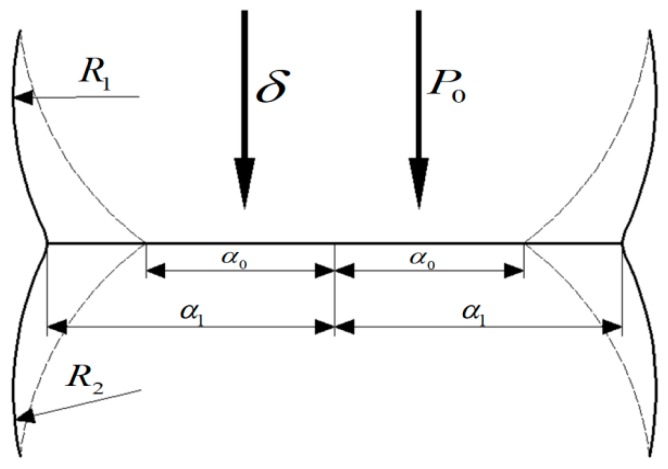
Schematic of interparticle contact in the Johnson–Kendall–Roberts (JKR) contact model theory.

The contact model is the core of the particle DEM. The JKR contact model [24,25,26] is an extension of the Hertz contact theory. The model is based on elastic solid contact and focuses on the “overlap” of contact between particles. The particle-matching ball mainly has a tangential elastic force, and the normal dissipative force and the tangential dissipative force interact. Two spherical particles are assumed to agglomerate only on the contact surface (Figure 7). When the external load is *P*_0_ and the elastic displacement is *δ*, the particle contact surface radius *α*_0_ can be obtained using the Hertz contact theory if the particles are not agglomerated. However, if the particles agglomerate, the contact surface radius *α*_1_ is greater than *α*_0_ although the external load is still *P*_0_.

In industrial DEM modeling, a method of amplifying the particle size and reducing the shear modulus is adopted because the number of particles may be in the order of several million, leading to a long calculation time; however, a scientific and reasonable scale basis is required. Thus, the accurate surface energy value, particle size, and mechanical properties should be coordinated in the JKR contact model. To achieve this, the concept of cohesion number [27] is proposed. The cohesion number is expressed as the ratio of cohesive work between particles to the gravitational potential energy of the particles.

(1) $$\mathrm{Cohesion}\;\mathrm{number}=\frac{\mathrm{Work}\;\mathrm{of}\;\mathrm{cohesion}}{\mathrm{Gravitational}\;\mathrm{potential}\;\mathrm{energy}}$$

This can be mathematically expressed as follows:

(2) $$C\mathrm{oh}=\frac{1}{\rho g}{\left(\frac{\gamma^{5}}{E^{{*}^{2}}R^{{*}^{2}}}\right)}^{\frac{1}{3}},$$

where r denotes the density of the particles, g denotes the acceleration of gravity, *γ* denotes the average surface energy of the particles, E* denotes the equivalent Young’s modulus of elasticity, and R* denotes the equivalent particle size from among various particles. The parameter R* is calculated according to Equation (3).

(3) $$R^{*}=(\frac{1}{R_{1}}+\frac{1}{R_{2}}{)}^{-1}{,E}^{*}=(\frac{{1-\mu }_{1}^{2}}{E_{1}}+\frac{{1-\mu }_{2}^{2}}{E_{2}}{)}^{-1},$$

where R_1_ and R_2_ are the radii of the contacting spheres, E_1_ and E_2_ are their Young’s moduli, and m_1_ and m_2_ are their Poisson’s ratios.

During the simulation, to avoid large overlaps in the process of contact between particles, which would increase the calculation cost, the cohesion number (Coh) is usually used to scale the particle discrete element model; further, the particle size is increased according to a certain zoom level. In addition, the equivalent surface energy is calculated by increasing the particle size and reducing the shear modulus. In addition, Alizadeh et al. [15] used this method. For example, the average surface energy of the PU particles is 0.0214 J/m^2^ [28]; when this value is substituted into Equation (2), the cohesion number is observed to be 0.000622. When the average surface energy value is maintained constant, the particle size is magnified 10 times. However, when the modulus is reduced by two orders of magnitude, the equivalent surface energy used in the simulation is 4.0 J/m^2^. Simultaneously, to accurately calibrate the calculated parameter values, we refer to the surface energy values of similar materials in the GEMM database provided by the EDEM software and expand the calibration range of the surface energy values with the equivalent surface energy value (4.0 J/m^2)^ as the intermediate value. Expanding the surface energy value to both sides, we determine the value between 3 and 8 J/m^2^.

#### 2.3.2. DEM Simulation Tests

**Figure 8:**
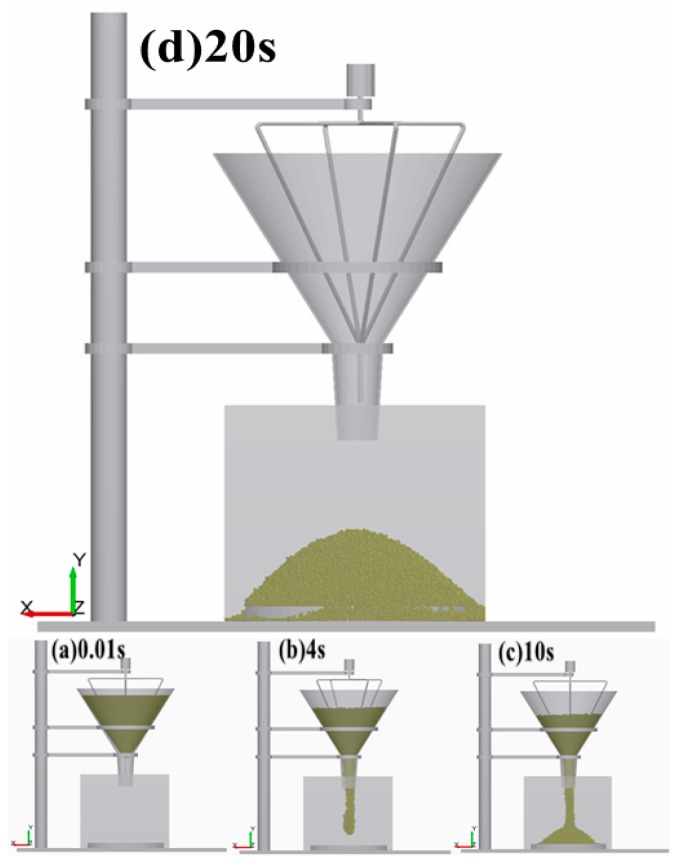
Process of the stacking angle simulation test: (**a**–**d**) simulation results corresponding to 0.01, 4, 10, and 20 s.

The discrete element virtual calibration test is completely consistent with the test device described in Section 2.1. Particle factory generation was compiled using the commercial DEM simulation software with an API open-source interface; the filling rate was 0.56, and the generation time was 0.01 s. The funnel was filled with the particle group, and the large moment between the particles was eliminated. After the particles were allowed to stand for 3 s, the brush was rotated at 60 rpm. At this time, the particle group fell freely, with a gravitational acceleration of 9.81 m/s^2^ (Figure 8), and finally formed a stable pile on the receiving table. The powder particle pile simulation time remained 20 s for the whole process, and the final 2 s was used for the excessive porosity when the particles piled up (this pile-up leads to the excessive formation of a simulated packing). The simulation test method based on that described in Section 2.3.1 was used to propose a coarse-grained modeling criterion based on the JKR model to characterize the actual accumulation phenomenon of the PU powder.

#### 2.3.3. Sensitivity Analysis of the Particle Intrinsic Parameters

To simplify the calibration process, we analyze the effect of the particle Eigen parameters on the angle of repose. Therefore, in combination with the range of values of the DEM simulation parameters shown in Table 1, the Eigen parameters (density, Young’s modulus, Poisson’s ratio, and particle aspect ratio) of the particles were investigated by the simulation test described in Section 2.3.2. Thus, we can conclude that the angle of repose was affected.

**Figure 9:**
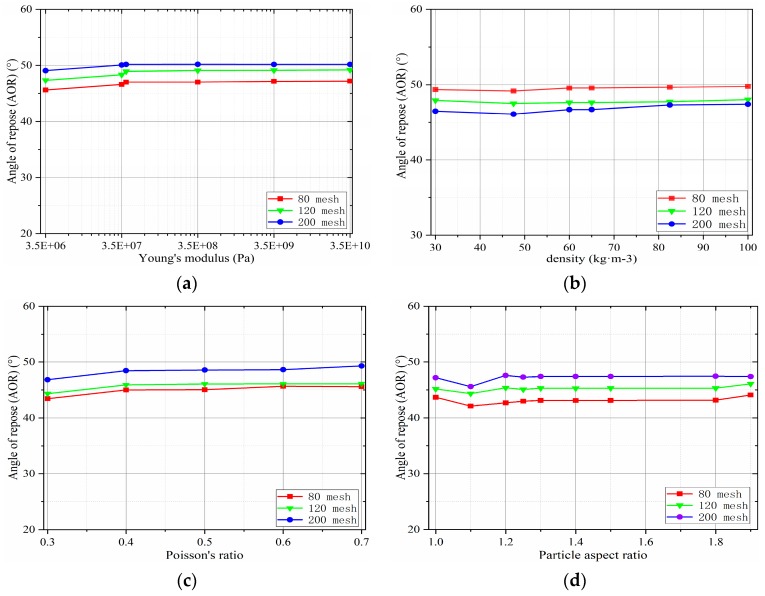
Particle intrinsic parameters: (**a**) Young’s modulus; (**b**) density; (**c**) Poisson’s ratio; and (**d**) particle aspect ratio.

Figure 9a denotes that the Young’s modulus is between $3.5\times 10^{6}$ and $3.5\times 10^{7}$ Pa and that the angle of repose gradually increases. Notably, almost no change is observed in the stacking angle when the Young’s modulus becomes greater than $3.5\times 10^{7}$ Pa. As described in Section 2.3.1, the computational time of discrete element simulation can be minimized by reducing the magnitude of Young’s modulus. In this case, the Young’s modulus is reduced by two orders of magnitude (set to $3.5\times 10^{7}$ Pa). The mid-rest angle has almost no effect. In addition, the particle density has little effect on the angle of repose (Figure 9b), consistent with the results obtained from other studies [29]. Similarly, the particle Poisson’s ratio increases from 0.3 to 0.7. Only a small reduction is observed in the static angle of repose of the particles (approximately 0.5). In the results of Bharadwaj et al. [23], the best aspect ratio problem for coarse particle granulation is proposed in Section 2.3.1, as depicted in Figure 9d. When the width ratio becomes 1.3, the angle of repose of the particles tends to be stable, consistent with the prediction of the static angle.

A set of thermodynamic PU powders with a particle size of 120 mesh (0.125 mm) was obtained through the aforementioned batch simulation test; the intrinsic parameters were Gp = $3.5\times 10^{7}$ Pa, $\rho_{P}$ = 60 kg·m^−3^, Vp = 0.4, particle aspect ratio = 1.3.

## 3. Development and Application of the BP Prediction Model

### 3.1. BP Neural Network Database

Multidimensional sampling of the calibrated discrete element contact parameters was performed using a uniform distribution-based LHS method [30] to form a virtual test sample space. The distribution function domain of each stochastic input variable is equally divided into $\Delta X_{i}^{k}(k=1,2,\ldots ,N)$ in probability, and each subinterval is subjected to independent equal-probability sampling. During each deterministic calculation analysis, sampling is strictly guaranteed in each subinterval. To ensure that the extracted random numbers belong to each subinterval, the random number $V_{i}$ in the *i*-th subinterval must satisfy the following conditions:

(4) $$V_{i}=\frac{V}{N}+\frac{i-1}{N},$$

(5) $$\frac{i-1}{N}<V_{i}<\frac{i}{N},$$

where *i* = 1, 2, …, *N*; *V_i_* is the random number of the first subinterval; and *V* is the random number with evenly distributed intervals. The LHS sampling design for n-dimensional random *X_i_* (*i* = 1, 2, …, *N*) variables involves the following two steps:

(1) Divide each random variable into N equal-probability subintervals $\Delta X_{i}^{k}(k=1,2,\ldots ,N)$, and obtain the midpoint of each subinterval as a sample representative $X_{i}^{k}$ of the corresponding random variable.
(2) For each random variable *X_i_*, extract a sample representative $X_{i}^{k}$ and arrange it according to a random number, and arrange the samples of all the random variables according to random numbers, resulting in *N* random arrangements. Each sample contains a sample of all the random variables representing $X_{i}^{k}$.

In this study, the sampling parameters e_pp_, *μ*_pp_, e_pw_, *μ*_pw_, and Γ, which were to be calibrated, were sampled at 120 sample points for BP neural network training and verification. Thus, all the sampling points constitute a 5 × 120 matrix. Each row of the matrix represents a variable, and each column represents a set of test parameters, with the first 80 samples denoting the training data and the final 40 denoting the validation data. The virtual test data are presented in Table A1 of Appendix A.

### 3.2. BP Neural Network Structure Design

**Figure 10:**
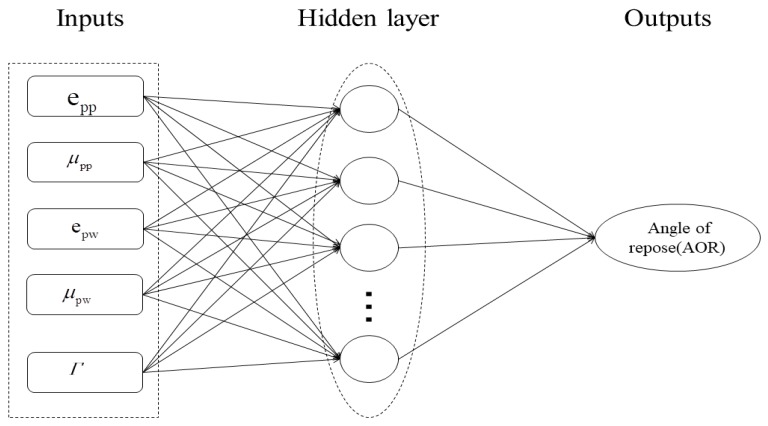
BP neural network topology.

The BP neural network was proposed by Rumelhart and McClelland [31]; it is a multilayer feedforward neural network trained according to the error BP algorithm. It is extensively used in many fields as a supervised learning algorithm that maps a set of inputs to the accurate output [32,33]. This study is based on the Sigmoid weighting function and uses the mean square error (MSE) deviation function as the core content of the BP neural network. In the current study related to discrete element parameter calibration, the DEM parameter is the general input layer, whereas the angle of repose is the output layer. Based on the DEM batch simulation experiment presented in Section 2.3.2, this study establishes an approximate model of the relation between the microscopic parameters and the macroscopic angle response for each set of sample points. The topological structure of the BP neural network prediction model for the particle macro parameter is presented in Figure 10. In addition, the numerical fluctuation of static angle is measured because of the defects of the BP neural network itself. Therefore, the GA is used to adjust the weight and threshold of the network to reduce the influence of noise in the model. Finally, the behavior of the particles can be predicted for any set of DEM simulation parameters. The BP neural network prediction model is subsequently used to extend the finite numerical simulation database to “infinity.”

### 3.3. Optimization of the BP Neural Network Performance on the Basis of the GA

**Figure 11:**
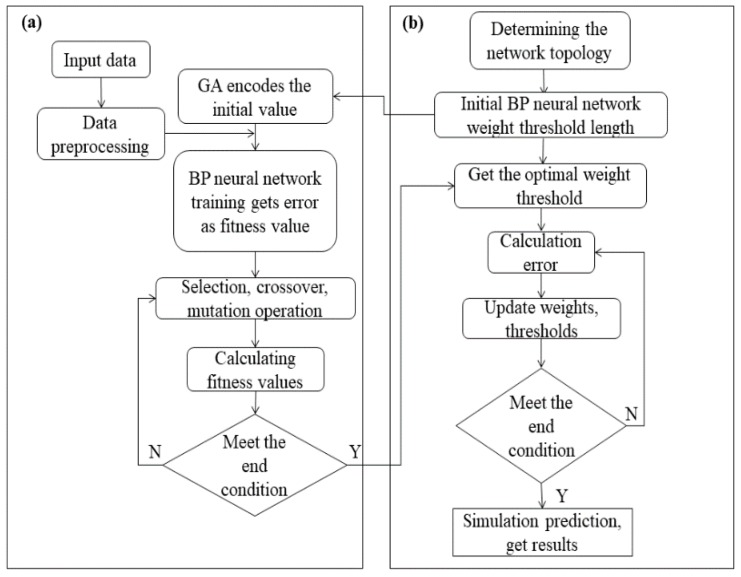
Flow chart for optimizing the BP neural network based on the genetic algorithm: (**a**) genetic algorithm (GA) part; (**b**) BP part.

While dealing with nonlinear problems, the BP neural network algorithm often converges to local solutions because of its serial search mechanism. To address this problem, the GA of “survival of the fittest in nature” is adopted [34]. Optimization to form a parallel search optimization method makes it easy to converge into the global sample space. While training the BP neural network, as depicted in Figure 11, the objective is to continuously optimize the weight and threshold of each layer such that the error between the final network output static angle value and the predicted output is sufficiently small in accordance with our expectations. The process of optimization is the process of constant “evolution” of weights and thresholds. During each iteration, the weights and thresholds are selected on the basis of the “natural environment,” mutations, and crossovers and then leave excellent varieties, i.e., weights and offsets minimize output errors. Inferior varieties are eliminated, and, after multiple iterations, the “genes” of weights and thresholds improve, achieving the training objectives. Furthermore, the GA-BP neural network is able to fit the data using fewer iterative steps. In this study, it can quickly find the input DEM parameters and optimize the target using the preset MSE values, thereby achieving global optimization faster.

In the BP neural network, the number of nodes in the input and output layers are determined, and the appropriateness of the number of nodes in the hidden layer is determined, which directly affects the performance of the network. In general, in case of a large sample space, a multilayer network is more accurate than a single-layer network; however, the time required for training considerably increases. An excessive number of hidden-layer nodes will increase the redundancy of the network, whereas the presence of very few hidden-layer nodes will increase the network error. No clear theoretical guidance is available for determining the number of hidden-layer nodes, which are generally determined by a combination of empirical formula methods and repeated test methods [35] and can be given as follows:

(6) $$h=\sqrt{m+n}+a,$$

where h is the number of hidden-layer nodes, m is the number of input layer nodes, and n is the number of output layer nodes, which is an adjustment constant between 1 and 10. The number of neurons in the hidden layer of the obtained Equation (6) is approximately 3–12, which is further explored below.

**Figure 12:**
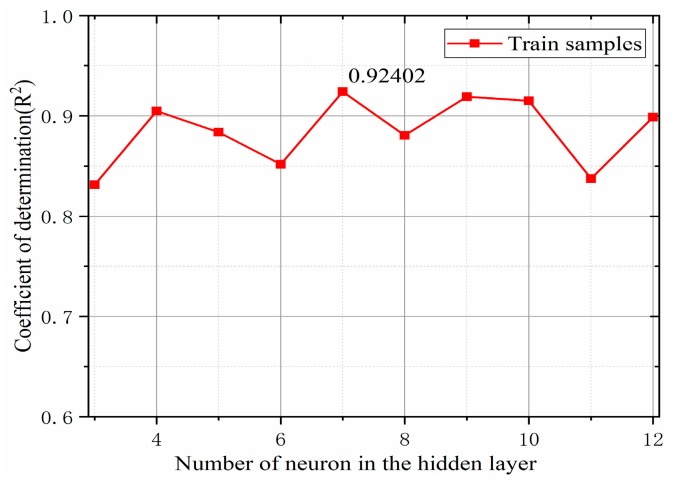
Implied layer node performance test.

Figure 12 denotes the relation between the number of neural network hidden-layer neurons determined by the BP neural network prediction model and the response-angle coefficient of determination R2. When the number of neurons in the hidden layer became seven, the coefficient of determination of the verification sample reached a maximum of 0.924. Therefore, seven hidden-layer neurons were selected to establish a BP neural network model for predicting the angle of repose.

### 3.4. Prediction Results and Discussion

**Figure 13:**
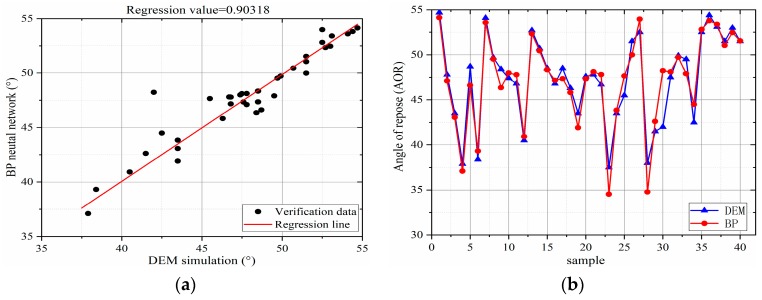
Accuracy analysis of the BP neural network after training: (**a**) 40 sets of verification parameter sets and (**b**) BP prediction values and DEM simulation response values.

Among the 120 sets of sample spaces sampled by Latin hypercube, 80 sets of data were used in the BP neural network training model, whereas the remaining 40 groups were used in the post-training BP model to analyze the fitting accuracy of the BP model to the DEM model. The training data and forecast data used in this study are presented in Table A1 of Appendix A. The fitting accuracy of the BP model was evaluated by the coefficient *R*^2^ determined in Equation (7). The closer the value of *R*^2^ is to 1, the more accurate will be the BP model. In general, the approximate model and *R*^2^ ≥ 0.9 are considered for engineering applications.

(7) $$R^{2}=1-\frac{\sum_{i=1}^{N}(y_{i}-{\hat{y}}_{i}{)}^{2}}{\sum_{i=1}^{N}(y_{i}-{\bar{y}}_{i}{)}^{2}},$$

where $y_{i}$ is the first simulated value of the model, ${\hat{y}}_{i}$ is the first predicted value of the model, ${\bar{y}}_{i}$ is the average of the simulated values, and *N* is the number of samples. The coefficient of determination *R*^2^ based on the stacking angle of Equation (7) is 0.93. Figure 13 presents a comparison between the predicted and simulated values.

As can be observed from Figure 13a, the evaluation coefficient is obtained as 0.924, which is calculated in accordance with Equation (7) by linearly fitting the relation between the DEM simulation value and the predicted value. However, Figure 13a also shows a small number of discrete points which can be attributed to the initial state and flow process of the sample powder particles to be tested during the simulation and to the poor reproducibility of the angle of repose caused by randomness. The predicted value of the BP model and the DEM simulation response angle are compared in Figure 13b. This discrete phenomenon can be observed only in a small amount, and the predicted value is close to the DEM simulation value, indicating that the established BP model is sufficiently reliable to be used for calibrating the DEM parameters.

### 3.5. Application of the BP Prediction Model

#### 3.5.1. BP Model Predictive Value of the Angle-of-Repose Search

**Figure 14:**
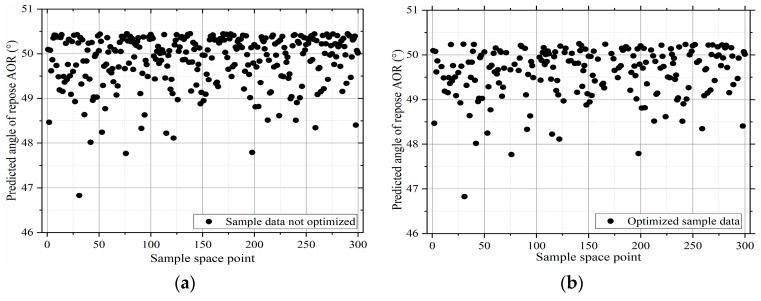
Spatial distribution of 300 samples: (**a**) unoptimized parameter set and (**b**) optimized parameter set.

To accurately calibrate the DEM parameters, the sample space was expanded to 300 sample points by LHS resampling and the mapping relation between the DEM parameters and the angle of repose was successfully established through the developed BP neural network (Figure 14a). The results denote a large dispersion and overlap in the output of the BP prediction model. To reasonably obtain a feasible solution that satisfies the mapping relation and to achieve a target value of the stacking angle mean of the actual test, which is listed in Appendix A, Table A2), the discrete outputs of the predicted output are compared and eliminated. The difference between the output value of the BP prediction model and that of the calibration experiment is less than 5% [19], and the condition is satisfied. These conditions can be expressed as:

(8) $${\mathrm{AOR}}_{Tol}=\mathrm{Object}\left|\frac{\mathrm{outputs}-\mathrm{targets}}{\mathrm{targets}}\right|\leq 5\%,$$

(9) Subject to outputs=f(inputs),

(10) $${\mathrm{inputs}}_{\min}\leq \mathrm{inputs}\leq {\mathrm{inputs}}_{\max}.$$

**Figure 15:**
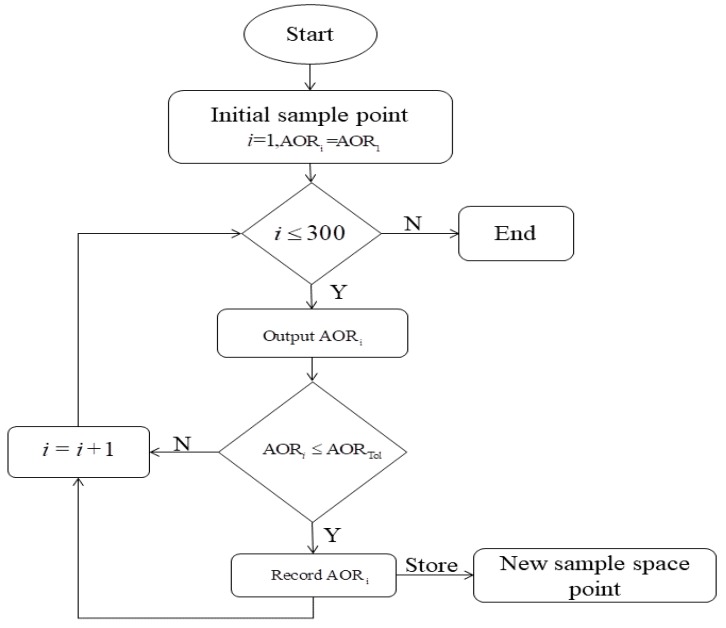
Flow of the search algorithm for predicting the angle of repose.

To quickly obtain a feasible solution that satisfies these conditions, a common loop structure algorithm is used to search for the distribution of the feasible domain space. The algorithm is presented in Figure 15.

The specific steps are as follows:

(1) initialize the sample point *i*, and predict the output value $AOR_{i}{=\mathrm{AOR}}_{1}$;
(2) identify whether the sample point *i* exceeds 300 sample points;
(3) filter the predicted values of the BP prediction model according to the AOR_Tol_ tolerance range and store them in a new sample space;
(4) repeat steps (2) and (3) until 300 sample points have been searched, resulting in a new sample space as depicted in Figure 14b.

#### 3.5.2. Mean-Shift Cluster Analysis

The mean-shift algorithm is a parameterless density estimation algorithm. The core idea of the mean-shift algorithm is to first calculate the offset mean of the current pixel, move the point to its offset mean, and continue to move as the starting point until the certain conditions are met and then stop iterating. Calculate the similarity between the cluster center candidate point and the candidate area sample, and use the maximum value of the similarity function to find the target mean-shift vector, move the cluster center candidate point to the offset mean, and iterate until the cluster center candidate point No offset occurs. In the DEM parameter optimization, a feasible domain may comprise multiple subdomains exhibiting similar solution values, indicating similar particle macroscopic properties. To effectively obtain the required DEM parameters, an appropriate algorithm is used to optimize the distribution and combination of feasible solutions. The classification and differentiation of data with approximate features using mean-shift clustering analysis [36] is a hill-climbing algorithm based on kernel density estimation, which is a clustering algorithm that does not require the number of clusters to be specified. Processing [37] and cluster analysis [38] are the commonly used cluster analysis methods in the field of data mining. The purpose of mean-shift clustering is to find the data sample points of the same cluster along the direction of increasing density. Further, the class with the highest frequency of sample point access is marked as the class of the current point set. The clustering results of this study for the optimized sample points (Figure 14b) are listed in Table 2.

### 3.6. Verification Instance

#### 3.6.1. DEM Parameter Verification Under Quasi-Static Conditions 

**Figure 16:**
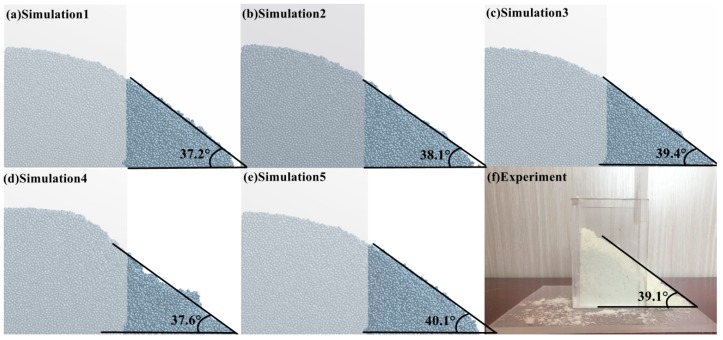
Simulation and verification tests of the baffle lifting method: (**a**–**e**) simulation results corresponding to 37.2°, 38.1°, 39.4°, 37.6°, and 40.1°; (**f**) the verification result is 39.1°.

Under quasi-static conditions, the verification of DEM input parameters is the most basic calibration method. The cumulative lift angle of polyurethane powder particles was verified by the baffle lift test commonly used for static angle of repose. The test apparatus is presented in Figure 16f; the square casing was made of 60 × 60 × 80 mm^3^ Plexiglas. During the test, a sample material with a mass of 15 g was placed in the square shell. When the baffle was lifted at a constant speed of 0.1 m/s, the flow of the PU powder resulted in the formation of a pile; after the particles of the population were stabilized, the formed powder particles were inclined and horizontal. The angle between the two is the static angle of repose of the powder. The simulation settings were consistent with the experimental conditions, and five sets of calibration parameters optimized in Section 3.5.2 were used as the inputs. The test simulation results are denoted in Figure 16a–e as 37.2°, 38.1°, 39.4°, 37.6°, and 40.1°, respectively. Surprisingly, it was found that the five sets of contact parameters under the dynamic conditions after calibration also apply to the static angle of repose and meet the AOR_Tol_ tolerances proposed in Section 3.5.1.

#### 3.6.2. Dynamic Condition DEM Parameter Verification

**Figure 17:**
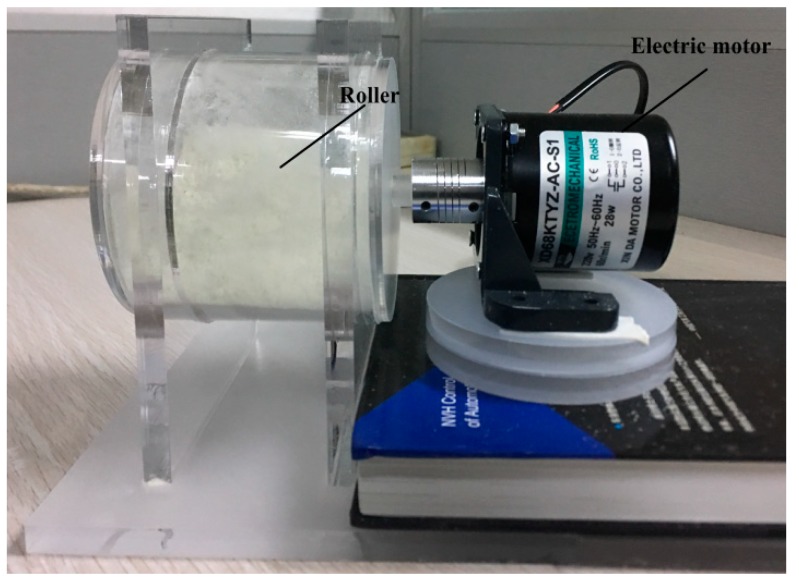
Rotary cylinder experiment device.

**Figure 18:**
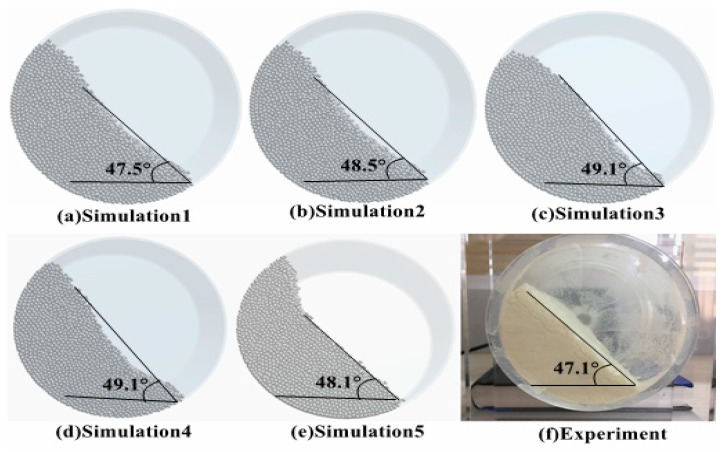
Simulation and verification tests for the rotating drum method: (**a**–**e**) simulation results corresponding to 47.5°, 48.5°, 49.1°, 49.1°, and 48.1°; (**f**) the verification result is 47.1°.

In order to further verify the DEM parameter calibration results, a more realistic dynamic angle of repose of the PU particles was verified via a rotary drum test. The experimental setup is shown in Figure 17. The cylinder is made of Plexiglas with an inner diameter and length of 45 and 100 mm, respectively. Prior to the experiment, the particle surface was set to a level with a particle loading of 30%, a rotational speed of 60 rpm, and a clockwise rotation for 5 s. After standing for 2 s, the excessive porosity was eliminated; after the particles were stabilized, the angle between the surface of the particles and the horizontal plane was measured as the dynamic angle of repose. The five sets of calibration parameters in Table 2 are used as inputs, and the simulation settings are consistent with the experimental conditions. The test results are shown in Figure 18. Small errors indicate the validity of the calibration results.

#### 3.6.3. Results and Discussion

In this section, mean-shift clustering analysis is used to obtain five sets of DEM parameter sets input into the baffle lift and drum device for dynamic and static angle verification, respectively. Table 3 gives the relative error between the response angles under different load conditions. It is found that the DEM parameter set measuring the dynamic angle of repose using the funnel flow test device in Section 2.1 does not meet the static angle of repose verification (22.18%, 20.29%, 17.57%, 21.34%, 16.11%). Such a large error is due to the fact that the powder is subjected to different conditions of the external load and the response-angle deviation is generated, which means that the material calibration test and verification examples under dynamic/static conditions need to be performed separately.

Whether under static (baffle lift test) or dynamic conditions (rotary drum test), the error between the comparison tests is lower than 5%, which is also in line with the error criteria mentioned by Ye et al. [19]. Simultaneously, we found that although the relative error verified by the baffle lift test is small, it is between 0.77% and 4.86%. However, the simulation angles (37.2°, 38.1°, 39.4°, 37.6°, 40.1°) are smaller than the average angle of repose of the batch funnel flow meter test (47.801°); instead, the response angles of the simulation from the rotary drum are 47.5°, 48.5°, 49.1°, 49.1°, and 48.1°, which are closer to the funnel flow meter test static angle. This shows that the accuracy of the DEM parameters of the PU powder calibration can be more clearly indicated via the rotary drum verification test.

## 4. Conclusions

The JKR model exhibiting adhesion characteristics was used for an ultrafine agglomerated powder (120-mesh PU powder) that is difficult to calibrate, and the sample material was characterized. A DEM model was used based on the improved FBS-104 powder measuring instrument. In the parameter calibration method, the GA–BP optimization algorithm was used during the calibration test to reduce the number of simulations required for optimization. The results of this study can be summarized as follows:

- To achieve the DEM parameter calibration of the micron-scale material samples, the concept of cohesion number proposed by Bharadwaj et al. [23] for coarse-grained discrete element modeling can improve the calibration efficiency without affecting the calibration accuracy.
- In the DEM parameter calibration process, the BP approximation model modified using the GA can easily converge into the global sample space to avoid local optimal solutions, which would result in large errors in the calibration results.
- The study found that the rotating drum as a verification example of dynamic conditions can be used to verify the DEM parameter set of the funnel flow meter more accurately than the baffle lift test under static conditions and that the relative error is 0.63%–2.72%.
- The intrinsic parameters of the particles were explored during the simulation. The small range of the intrinsic parameters of the particles did not strongly influence the angle of repose; however, reducing the shear modulus was observed to considerably reduce the calculation cost.
- To avoid large dispersion and overlap between the predicted value and the simulated target value, a combination of the cyclic search algorithm and mean-shift cluster analysis can be used to find the optimal solution for each subclass in the sample space.
- Although the ultrafine agglomerated PU powder exhibited poor reproducibility with respect to the angle of repose during the stacking test, the baffle lifting method verified that the predicted output value of the BP model after training agreed with the angle obtained in the actual stacking test.
- During this calibration process, we observed that the DEM parameter calibration results are not a set of solution sets; each output value (angle) has multiple sets of mapping relations with the input DEM parameters. This also revealed the calibration work of the DEM parameter, which is a search process of the parameter set “best combination.” Based on a test design method, AOR was used as the target response value to find a suitable mapping relation for application in this study.
- In the future, a large number of variables (bulk density, time step) can be added as the response value of the output layer of the prediction model, and the influence of the DEM parameters on the angle of repose can be further investigated.

## Figures and Tables

**Table 1:** Discrete element method (DEM) parameters, relevant symbols, and values/intervals used in this study; parameters to be calibrated are set to intervals, which are marked with ★.

DEM Parameter	Symbol	Value/Interval
Polyurethane Poisson’s ratio	$V_{p}$	0.35–0.39
Polyurethane Young’s modulus (Pa)	$G_{P}$	$3.5\times 10^{9}$
Polyurethane density (kg·m^−3^)	$\rho_{P}$	30–100
Poisson’s ratio of steel	$V_{w1}$	0.3
Young’s modulus of steel (Pa)	$G_{w1}$	$1.0\times 10^{9}$
Steel density (kg·m^−3^)	$\rho_{w1}$	7850
Poisson’s ratio of Plexiglas	$V_{w2}$	0.4
Shear modulus of Plexiglas (Pa)	$G_{w2}$	$1.6\times 10^{10}$
Density of Plexiglas (kg·m^−3^)	$\rho_{w2}$	1385
Collision recovery coefficient (particle–particle)	$e_{pp}$	★0.1–0.5
Static friction coefficient (particle–particle)	$\mu_{pp}$	★0.3–0.75
Rolling friction coefficient (particle–particle)	$\mu_{pp}^{\gamma }$	0.001
Collision recovery coefficient (particle–wall)	$e_{pw}$	★0.1–0.6
Static friction coefficient (particle–wall)	$\mu_{pw}$	★0.5–2.5
Rolling friction coefficient (particle–wall)	$\mu_{pw}^{\gamma }$	0.001
JKR surface energy (J/m^2^)	*Γ*	★3–8

**Table 2:** Results of mean-shift cluster analysis.

Group	Calibrated DEM Parameters				
	$e_{\mathrm{pp}}$	$\mu_{\mathrm{pp}}$	$e_{\mathrm{pw}}$	$\mu_{\mathrm{pw}}$	$\Gamma ({J/m}^{2})$
1	0.320426	0.353256	0.490065	0.930533	5.974576
2	0.267255	0.371349	0.565715	0.670134	4.38862
3	0.295006	0.327425	0.506216	1.734846	4.818618
4	0.301659	0.350872	0.537846	1.660376	3.609546
5	0.262054	0.336516	0.519476	0.792432	7.318412

**Table 3:** Relative error between response angles under dynamic/static load conditions.

	Group 1	Group 2	Group 3	Group 4	Group 5
Simulation of rotating drum/(°)	47.5	48.5	49.1	49.1	48.1
Rotating drum test/(°)	47.1				
Rotating drum contrast test (relative error)	0.85%	2.97%	4.25%	4.25%	2.12%
Simulation of baffle lift/(°)	37.2	38.1	39.4	37.6	40.1
Baffle lift test/(°)	39.1				
Baffle lift contrast test (relative error)	4.86%	2.56%	0.77%	3.84%	2.56%
Funnel flow meter test/(°)	47.801				
Funnel flow meter test and baffle lift simulation (relative error)	22.18%	20.29%	17.57%	21.34%	16.11%
Funnel flow meter test and rotary drum simulation (relative error)	0.63%	1.46%	2.72%	2.72%	0.63%

## Appendix A

**Table A1:** BP neural network database.

$e_{\mathrm{pp}}$	$\mu_{\mathrm{pp}}$	$e_{\mathrm{pw}}$	$\mu_{\mathrm{pw}}$	$\Gamma \;({J/m}^{2})$	AOR (°)	Predicted value AOR (°)
0.148014	0.355076	0.135264	0.736817	0.736817	37.5	-
0.312023	0.537557	0.258614	1.551627	1.551627	43.2	-
0.327493	0.555877	0.270874	1.641178	1.641178	46.5	-
0.446438	0.689714	0.358998	2.222321	2.222321	52.2	-
0.141479	0.34653	0.130286	0.711667	0.711667	38.5	-
0.304733	0.531433	0.25321	1.52806	1.52806	46.5	-
0.16831	0.378255	0.152086	0.849553	0.849553	38.5	-
0.126852	0.331476	0.12139	0.649442	0.649442	38.5	-
0.423007	0.663505	0.341825	2.115487	2.115487	52.5	-
0.121281	0.323449	0.115855	0.604728	0.604728	36.5	-
0.333261	0.56006	0.272745	1.657726	1.657726	46.6	-
0.21847	0.432949	0.187752	1.096404	1.096404	44.5	-
0.241529	0.458257	0.205444	1.213885	1.213885	45.5	-
0.107443	0.308152	0.105226	0.545632	0.545632	33.7	-
0.212468	0.425678	0.183951	1.053621	1.053621	42.5	-
0.418222	0.65724	0.338593	2.095684	2.095684	50.9	-
0.276442	0.497534	0.231542	1.372128	1.372128	48.5	-
0.423445	0.667499	0.344465	2.128864	2.128864	51.5	-
0.319158	0.546443	0.262997	1.586359	1.586359	47.5	-
0.288473	0.512738	0.2408	1.444792	1.444792	47.5	-
0.296496	0.519416	0.246191	1.468489	1.468489	48.6	-
0.207081	0.420897	0.18249	1.038653	1.038653	44.5	-
0.299113	0.524424	0.247849	1.495777	1.495777	47.5	-
0.374274	0.610314	0.305546	1.878514	1.878514	48.8	-
0.153651	0.360635	0.14243	0.782299	0.782299	37.5	-
0.117663	0.32115	0.113377	0.589483	0.589483	35.5	-
0.151342	0.358549	0.138751	0.763457	0.763457	38.5	-
0.200513	0.412871	0.175436	1.01327	1.01327	43.2	-
0.272885	0.492047	0.228874	1.3599	1.3599	48.5	-
0.447476	0.690637	0.3602	2.243708	2.243708	53	-
0.462514	0.708098	0.371081	2.30172	2.30172	53.2	-
0.49869	0.747369	0.397784	2.498688	2.498688	54.9	-
0.408619	0.648391	0.330753	2.043503	2.043503	50.4	-
0.229198	0.443187	0.195137	1.144559	1.144559	46.3	-
0.124055	0.327711	0.117612	0.624993	0.624993	37.5	-
0.391213	0.627576	0.319341	1.959905	1.959905	50.6	-
0.468248	0.714486	0.376739	2.335572	2.335572	54.5	-
0.367979	0.60188	0.30052	1.838435	1.838435	48.4	-
0.146516	0.350077	0.134187	0.722417	0.722417	38.5	-
0.471483	0.717974	0.379428	2.364548	2.364548	53.6	-
0.221485	0.43738	0.191663	1.114118	1.114118	43.6	-
0.135678	0.340273	0.125432	0.679225	0.679225	36.8	-
0.48366	0.732491	0.387561	2.425036	2.425036	54.2	-
0.377803	0.61492	0.309358	1.88945	1.88945	48.8	-
0.188603	0.400831	0.165758	0.947453	0.947453	41.4	-
0.452653	0.694399	0.363694	2.255973	2.255973	52.6	-
0.25078	0.470105	0.21425	1.25443	1.25443	48.6	-
0.380085	0.617621	0.312161	1.908359	1.908359	49.1	-
0.433769	0.678038	0.351069	2.17868	2.17868	51.7	-
0.306967	0.533354	0.256798	1.544442	1.544442	45.4	-
0.23591	0.450782	0.201923	1.181499	1.181499	47.5	-
0.224564	0.442093	0.192686	1.117348	1.117348	45.8	-
0.16351	0.374564	0.148299	0.817834	0.817834	39.5	-
0.302886	0.52835	0.251428	1.513066	1.513066	48.3	-
0.131847	0.336389	0.124413	0.655122	0.655122	35.5	-
0.23827	0.456382	0.202916	1.189952	1.189952	47.5	-
0.260284	0.481961	0.221172	1.305584	1.305584	46.7	-
0.254459	0.475788	0.215289	1.27702	1.27702	47.5	-
0.441856	0.685963	0.355206	2.206748	2.206748	51.5	-
0.32147	0.550521	0.266212	1.60286	1.60286	46.5	-
0.324843	0.552813	0.269607	1.623838	1.623838	46.3	-
0.18121	0.390041	0.160593	0.909805	0.909805	41.5	-
0.162969	0.369847	0.145247	0.803433	0.803433	38.4	-
0.19726	0.411867	0.173669	0.994007	0.994007	44.5	-
0.340307	0.573351	0.281061	1.707059	1.707059	47.1	-
0.170813	0.381489	0.153139	0.851311	0.851311	39.5	-
0.104023	0.307472	0.103119	0.526815	0.526815	31.8	-
0.387796	0.625467	0.317023	1.94451	1.94451	49.4	-
0.385571	0.619061	0.314785	1.926352	1.926352	49.3	-
0.410039	0.65005	0.333638	2.052398	2.052398	50.5	-
0.370615	0.604706	0.304686	1.852664	1.852664	48.6	-
0.282934	0.506236	0.236888	1.41209	1.41209	48.7	-
0.475776	0.723432	0.381433	2.371023	2.371023	53.7	-
0.102568	0.300973	0.101261	0.516099	0.516099	31.3	-
0.401425	0.640962	0.326776	2.003317	2.003317	50.4	-
0.432507	0.672806	0.347558	2.150266	2.150266	51.6	-
0.29331	0.516569	0.244004	1.465484	1.465484	47.9	-
0.361881	0.592732	0.297125	1.807417	1.807417	48.4	-
0.177314	0.389413	0.159907	0.886797	0.886797	39.4	-
0.259868	0.477258	0.218398	1.291127	1.291127	48.7	-
0.496221	0.742673	0.395921	2.48252	2.48252	54.7	54.1343
0.263487	0.487348	0.223437	1.31955	1.31955	47.8	47.09626
0.195801	0.408062	0.170152	0.983121	0.983121	43.5	43.07308
0.13885	0.342139	0.129412	0.694485	0.694485	37.9	37.10446
0.248251	0.468067	0.210299	1.239911	1.239911	48.7	46.61237
0.157703	0.365908	0.143041	0.798988	0.798988	38.4	39.31297
0.483311	0.729671	0.387418	2.409156	2.409156	54.1	53.59277
0.394428	0.632054	0.321426	1.975155	1.975155	49.7	49.52709
0.246637	0.461935	0.209964	1.220124	1.220124	48.4	46.35139
0.347334	0.580223	0.28592	1.737206	1.737206	47.4	47.99007
0.336898	0.566945	0.277764	1.684021	1.684021	46.8	47.7934
0.1753	0.383506	0.156862	0.876854	0.876854	40.5	40.91912
0.454227	0.697562	0.367317	2.278103	2.278103	52.7	52.33081
0.41565	0.65385	0.336594	2.068696	2.068696	50.7	50.44045
0.363435	0.596835	0.29987	1.833117	1.833117	48.5	48.34836
0.267718	0.488204	0.225227	1.33657	1.33657	46.8	47.16393
0.278919	0.500717	0.233969	1.388731	1.388731	48.5	47.34501
0.232639	0.448623	0.198307	1.166255	1.166255	46.3	45.81617
0.185732	0.396439	0.164037	0.919877	0.919877	43.5	41.91603
0.283575	0.507758	0.239619	1.417782	1.417782	47.6	47.3336
0.35404	0.58545	0.290478	1.772903	1.772903	47.8	48.11718
0.335475	0.565447	0.275944	1.672342	1.672342	46.7	47.80509
0.11331	0.314101	0.108047	0.558355	0.558355	37.5	34.5128
0.205706	0.417912	0.17927	1.032569	1.032569	43.5	43.83734
0.31594	0.542452	0.260678	1.566943	1.566943	45.5	47.65645
0.405597	0.642057	0.328437	2.026057	2.026057	51.5	49.98852
0.490435	0.74106	0.394663	2.464506	2.464506	52.5	53.97321
0.115208	0.317845	0.110801	0.572423	0.572423	38	34.75902
0.192786	0.404665	0.167635	0.95128	0.95128	41.5	42.62196
0.358329	0.590099	0.293082	1.790835	1.790835	42	48.23795
0.352469	0.58337	0.287791	1.764129	1.764129	47.5	48.10753
0.398763	0.637235	0.322779	1.988032	1.988032	49.9	49.7124
0.344042	0.57628	0.284129	1.718395	1.718395	49.5	47.90788
0.216065	0.427818	0.187274	1.070227	1.070227	42.5	44.48258
0.464179	0.709876	0.3729	2.331597	2.331597	52.5	52.80379
0.48677	0.735263	0.391804	2.44262	2.44262	54.4	53.80025
0.478557	0.724111	0.384023	2.388136	2.388136	53.1	53.39367
0.427445	0.668181	0.345401	2.139559	2.139559	51.5	51.03914
0.457369	0.701879	0.369677	2.284386	2.284386	53	52.46019
0.438178	0.678875	0.354788	2.185601	2.185601	51.5	51.52571

**Table A2:** Batch funnel flow meter test.

Test Sequence	Simulation AOR (°)	Test Sequence	Simulation AOR (°)	Test Sequence	Simulation AOR (°)
1	45.5	35	50.5	69	48.4
2	46.4	36	47	70	48.3
3	47.4	37	48.1	71	48.9
4	48.3	38	49.4	72	46.7
5	47.2	39	46.8	73	48.6
6	46.3	40	48.6	74	47.2
7	47.5	41	49.5	75	47.2
8	43.6	42	47.5	76	48.3
9	45.8	43	47.9	77	45.6
10	48.3	44	50.1	78	46.9
11	49.2	45	48.2	79	48.6
12	48.4	46	47.2	80	48.5
13	49.2	47	48.3	81	48.5
14	47.5	48	45.4	82	47.8
15	46.8	49	48.1	83	48.4
16	49.5	50	50.1	84	48.3
17	49.5	51	49.4	85	48.5
18	46.3	52	50.2	86	48.5
19	48.9	53	46.3	87	46.9
20	47.3	54	48.3	88	49.2
21	48.9	55	46.8	89	47.7
22	48.8	56	47.6	90	48.2
23	47.9	57	48.4	91	49.3
24	47.1	58	47.4	92	47.9
25	46.5	59	45.2	93	49.6
26	45.9	60	44.5	94	48.5
27	48.2	61	47.9	95	49.4
28	45.6	62	48.9	96	41.2
29	49.2	63	47.1	97	40.5
30	48.5	64	47.5	98	49.8
31	46.8	65	48.9	99	49.9
32	45.8	66	48.9	100	48.8
33	49.5	67	48.4		
34	50.2	68	47.8		
The average value of the static angle of the bulk accumulation experiment is 47.801°.					
